# Down-regulation of STAT3 induces the apoptosis and G1 cell cycle arrest in esophageal carcinoma ECA109 cells

**DOI:** 10.1186/s12935-018-0549-4

**Published:** 2018-04-04

**Authors:** Chao Zhou, Jie Ma, Mengyuan Su, Dan Shao, Jianan Zhao, Tongjian Zhao, Zhuoyao Song, Yan Meng, Ping Jiao

**Affiliations:** 1 School of Pharmaceutical Sciences, Jilin University, 1266 Fujin Road, Changchun, Jilin 130021 People’s Republic of China; 2 The First Hospital of Jilin University, 1163 Xinmin Street, Changchun, Jilin 130021 People’s Republic of China

**Keywords:** Apoptosis, Esophageal cancer, STAT3, shRNA

## Abstract

**Background:**

Signal transducer and activator of transcription 3 (STAT3) is persistently activated in a wide variety of epithelial cancers. Aberrant activity of STAT3 correlates with tumor growth, invasion and metastasis, which makes it a potential therapeutic target of cancer. To explore the biological role of STAT3 in esophageal cancer, we used small hairpin RNA to knockdown the expression of the STAT3 gene in the esophageal carcinoma ECA109 cell line and the cell apoptosis, cell cycle and cell migration were investigated.

**Methods:**

The cell apoptosis was tested using DNA ladder, mitochondrial membrane potential assay, TUNEL assay, annexin V-PI staining. Cell cycle phases were estimated using flow cytometry analysis. The mRNA and proteins related to apoptosis and cell cycle were examined by quantitative real-time polymerase chain reaction (qRT-PCR) and Western blot, respectively. And cell migration was investigated by in vitro Transwell assay. The data were analyzed with two-sample Student’s t test and ANOVA followed by the LSD post hoc test.

**Results:**

Our results showed that knockdown of STAT3 in ECA109 cells induced noticeable apoptotic morphological changes like cell shrinkage, apoptotic vacuoles, membrane blebbing time-dependently. In addition, DNA ladder, TUNEL assay, Annexin V-PI staining and declined level of cleaved Caspase-3 indicated that down-regulation of STAT3 could induce apoptosis in ECA109 cells. Flow cytometry analysis displayed the induction of G1-phase cell cycle arrest of ECA109 cells by STAT3 decreasing, consistent with the descend of c-Myc and cyclin D1 in protein levels. Furthermore, STAT3 knockdown suppressed the expression of matrix metalloproteinases-9, sushi domain containing 2 and urokinase plasminogen activator in ECA109 cells and inhibited cell migration ability.

**Conclusions:**

Knockdown of STAT3 could induce the apoptosis and G1 cell cycle arrest in esophageal carcinoma ECA109 cells, and inhibit the migration ability of cells as well.

## Background

Esophageal cancer, a common malignant gastrointestinal tumor, is the sixth leading cause of cancer death worldwide, with nearly 400,000 deaths and 500,000 new cases of esophageal cancer are diagnosed yearly. In spite of the use of modern surgical techniques combined with various treatment strategies, such as radiotherapy and chemotherapy, the prognosis of esophageal cancer is still poor, with the overall 5-year survival rates are less than 20% [1–4]. Thus, early diagnosis and potential new therapeutic targets are highly needed.

Signal transducers and activators of transcription (STATs) consist of an important transcription factor family that mediates fundamental function signal transmissions from cell surface to nucleus. STATs were first described in 1994 as members of the Janus kinase (JAK)-STAT pathway, in the context of interferon-α(INFα)-INFγ and interleukin-6 (IL-6)-mediated downstream signaling [5–7]. As an important member of STATs, STAT3 is considered to be involved in the regulation of a variety of critical functions, including cell survival, differentiation, cell cycle, angiogenesis, apoptosis and metastasis [8–11]. The potential oncogenic role of STAT3 was established by the expression of constitutively activated STAT3 in various tumor cell lines including breast, melanoma, pancreas, prostate, colon and esophagus and stomach [12–15]. Several cytokines and growth factors like IL-6 or epidermal growth factor (EGF) family members, as well as hepatocyte growth factor (HGF)contribute to the phosphorylation of STAT3, then the active STAT3 interacts with its target gene to prevent apoptosis [16] and stimulates transcription of key cancer genes linked with cell cycle [17]. To sum up, the STAT3 is a potential therapeutic target for cancer. In the present study, we targetedly down-regulated the expression of STAT3 in esophageal cancer ECA109 cell line using STAT3-specific shRNA vector pSi-STAT3 plasmid and investigated the effects of STAT3 down-regulation on ECA109 cells.

## Methods

### Materials and reagents

The human esophageal carcinoma ECA109 cell line was obtained from ATCC (Manassas, VA, USA). Plasmid pSi-STAT3 is a human STAT3-specific shRNA expression vector and pSi-Scramble is a negative control plasmid. The plasmids were kindly provided by Dr. Xuejian Zhao (Jilin University, Jilin, China). Dulbecco’s modified Eagle’s medium (DMEM) and fetal bovine serum (FBS) were obtained from the Hyclone (Logan, UT, USA). Opti-MEM reduced serum medium and Lipofectamine 2000 transfection reagent were purchased from Invitrogen Life Technology (Carlsbad, CA, USA). Annexin-V-FLUOS staining kit and complete protease inhibitor cocktail tablets were purchased from Roche Diagnostics (Indianapolis, IN, USA). TUNEL assay kit was from Keygen Biotechnology (Beijing, China). Mitochondrial membrane potential assay kit with JC-1 and DNA ladder extraction kit were purchased from Beyotime Institute of Biotechnology (Haimen, JiangSu, China). Antibodies to GAPDH, c-Myc and horseradish peroxidase-labelled conjugated secondary antibodies to rabbit, mouse and goat primary antibodies were purchased from Santa Cruz Biotechnology (Santa Cruz, CA, USA). Antibodies against STAT3 and Caspase-3 (32 kDa) were purchased from Cell Signaling Technology (Danvers, MA, USA). Primary antibodies of Cyclin D1, cleaved Caspase-3 (17 kDa) and enhanced chemiluminescence reagent were purchased from Pierce Biotechnology (Rockford, IL, USA). Transwell chambers were purchased from Corning Incorporated (New York, USA). RT-PCR was performed using the TaKaRa RNA PCR kit from Takara Biotechnology (Dalian, Liaoning, China). All other chemicals were analytical reagent grade.

### Cell culture and treatments

The ECA109 cell line was maintained in DMEM with 10% (v/v) FBS at 37 °C, 5% CO_2_. The cells were passaged around every 3 days. When cells grew to 90% confluence, they were digested and plated into 6-well plates and cultured for 14 h to get the 75% confluence before transfection. The ECA109 cells were transfected with plasmids of pSi-STAT3 or pSi-Scramble, respectively, using Lipofectamine 2000 according to the manufacturer’s instructions for 24–72 h before being analysed.

### Mitochondrial membrane potential assay

The change of the mitochondrial membrane potential (∆ψm) implies the early stage apoptosis. In this study, the alterations in ∆ψm were monitored under fluorescence microscopy using JC-1 dye. After transfected with pSi-STAT3 and pSi-Scramble for 24 h, the ECA109 cells were incubated with JC-1 for 20 min at 37 °C, 5% CO_2_. After the incubation, the dye was removed and the cells were washed twice with JC-1 buffer and examined with a fluorescent microscope using both red and green channels.

### DNA fragmentation assay

The ECA109 cells were transfected with pSi-STAT3 or pSi-Scramble, respectively, for 24 h, then harvested and washed with PBS buffer. The genomic DNA was extracted according to the manufacturer’s protocol of DNA ladder extraction kit. DNA samples were subsequently examined with 2% (w/v) agarose gel electrophoresis. The gel was visualized using ethidium bromide staining under ultraviolet light.

### TUNEL assay

The ECA109 cells transfected with plasmids were processed for TUNEL assays with one step apoptosis in situ assay kit. Briefly, cells were plated onto the slides, fixed with 4% paraformaldehyde for 30 min, and then washed with PBS buffer, incubated in TdT buffer containing fluorescein-dUTP for 1 h at 37 °C. Then cells were checked under fluorescence microscope. The nuclei of normal cells are stained in blue, while the apoptotic cells are red.

### Analysis of apoptosis by annexin V-FLUOS assay

The effect of pSi-STAT3 on ECA109 cell apoptosis was examined using annexin V-fluorescein and propidium iodide (PI) according to the manufacturer’s manual. Briefly, ECA109 cells transfected with or without plasmids were collected after trypsinized and washed with PBS twice, resuspended with 100 μl annexin V-FLUOS labeling solution and incubated for 15 min at 25 °C. A flow cytometry system was used to analyze the apoptotic ECA109 cells. The data were analyzed using Cell Quest data acquisition and analysis software (BD, Franklin Laked, NJ, USA).

### Flow cytometry analysis of cell cycle

Distribution of ECA109 cells in various cell cycle phases was estimated using a flow cytometer by quantitation of DNA content of cells stained with PI. The ECA109 cells were harvested by trypsinization after transfected with the plasmids, then washed with chilled PBS containing 4 mmol/l EDTA, and fixed with 70% cold ethanol at 4 °C for 30 min. After fixation, the cells were collected by centrifugation and washed with PBS. Then the pellets were resuspended and incubated in PBS containing PI, Triton X-100 and RNaseA at 4 °C for at least 30 min. The cell cycle phase distribution was assessed by flow cytometry.

### Cell migration assay

Cell migration was determined using transwell chambers. The ECA109 cells were transfected with plasmids for 72 h, then harvested with trypsinization. 1 × 10^5^ cells were plated in 200 μl serum-free DMEM medium on the top chamber, while 400 μl DMEM containing 10% FBS were plated in the lower chamber. After 14 h of incubation, the cells on the top surface were removed. The chambers were washed with PBS three times and the cells on the bottom surface were fixed in 4% paraformaldehyde for 15 min. After fixing at room temperature, the chambers were rinsed in PBS and stained with 0.2% crystal violet for 15 min. Then, the cells on the top chamber were removed, and the cells on the lower chamber were counted under the microscope.

### RNA extraction and quantitative real-time PCR

For RNA extraction, the ECA109 cells were transfected with plasmids for 48 or 72 h in the 12-well plates. Cells were washed with PBS before 500 μl Trizol reagent was added, and collected into the tubes then put on ice in the Trizol for 5 min. Subsequently, 100 μl chloroform was added, and the cells were heavily shaked for 30 s, put at room temperature for 10 min. Then the samples were centrifuged at 12,000*g* for 15 min resulting in two phases. Following centrifugation, the upper layer of supernatant was collected and added equal volume of isopropanol. The samples were stored on ice for 10 min and then centrifuged at 12,000*g* for 30 min at 4 °C. The RNA pellet was washed with 75% ethanol twice and centrifuged at 12,000*g* for 5 min. The isolated RNA was air-dried and dissolved in DEPC treated water, and reversely transcripted to cDNA using primescript™ RT reagent kit. Real-time PCR was performed with SYBR^®^premix ex Taq™ II, ROX plus reagent kit, conducted in step one plus™ real-time PCR system (Thermo Fisher Scientific, Waltham, MA, USA). The PCR program was initiated at 94 °C for 10 min, followed by 40 cycles of 90 °C 5 s, 60 °C 30 s, products were verified by melting curve analysis. The results were normalized to GAPDH and were calculated from threshold cycle numbers. Fold-changes in target gene mRNA expression were determined using ΔΔCt method. The same calculation formula as determined in the microarray analysis. The fold induction = 2^−ΔΔCt^, where Ct is the threshold cycle number, and ΔΔCt = [Ct gene of interest (unknown sample) − Ct GAPDH (unknown sample)] − [Ct gene of interest (calibrator sample) − Ct GAPDH (calibrator sample)]. Sequences of the primers used for the test were as follows: MMP-9: forward, 5′-ACCTGGGCAGATTCCAAACCT-3′; reverse, 5′-CGGCAAGTCTTCCGAGTAGT-3′. uPA: forward, 5′-GAGAATTCACCACCATCGA-3′; reverse, 5′-GCTGCCTCCACACACGTAG-3′. SUSD2: forward, 5′-TCACTGGACAACGGCCAC-3′; reverse, 5′-CGTAGTATTGCCAACGCGTC-3′. GAPDH: forward, 5′-GCACCACCAACTGCTTAG-3′; reverse, 5′-GCAGGGATGATGTTCTGG-3′.

### Western blot analysis

For Western blot analysis, the ECA109 cells were washed with ice-cold PBS and lysed with ice-cold lysis buffer (1% Triton X-100, 50 mmol/l HEPES, 50 mmol/l sodium pyrophosphate, 100 mmol/l sodium fluoride, 10 mmol/l EDTA, 10 mmol/l sodium vanadate) containing protease inhibitors cocktail on ice. After centrifugation at 15,000*g* for 15 min at 4 °C, the supernatant was analyzed for protein content using BCA protein assay kit. The protein was heated at 100 °C for 5 min, and a total of 60 μg protein was separated on 8–15% sodium dodecyl sulfate polyacrylamide (SDS-PAGE) gels, then transferred onto a PVDF membrane. The membranes were blocked with 5% milk in TBST buffer at room temperature for 1 h and were incubated with the primary antibodies at 4 °C overnight. After the membranes were washed three times with TBST buffer, they were incubated with a corresponding secondary antibody in TBST buffer for 1 h at room temperature, followed by washing three times with TBST. The protein-antibody bound bands were visualized using ECL reagents and the signal strength of each protein was normalized against the corresponding control.

### Statistical analysis

Values are presented as the mean ± standard errors (SE). Data analysis for comparison between treated groups and corresponding controls was performed using SPSS software (IBM, Armonk, NY, USA), and the data were analyzed with two-sample Student’s t test and ANOVA followed by the LSD post hoc test. P < 0.05 was considered to be statistically significant.

## Results

### Inhibition of STAT3 expression in ECA109 cells through plasmid-based shRNA

**Fig. 1:**
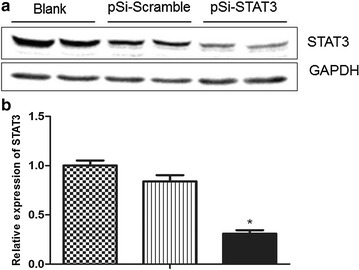
Verification of the knockdown efficiency of STAT3 in ECA109 cells. **a** Western blot analysis of STAT3 protein levels of ECA109 cells transfected with pSi-STAT3 or pSi-Scramble and untreated cells. GAPDH was used as a loading control. **b** Quantity of STAT3 protein levels compared to GAPDH. Data are represented as the mean ± SE, *P < 0.05, pSi-STAT3 group vs. pSi-Scramble group

To investigate the biological functions of STAT3 downregulation, we utilized recombinant plasmid of shRNA to inhibit endogenous STAT3 in ECA109 cells. The pSi-STAT3 plasmid was specifically against human STAT3 and pSi-Scramble was as a control plasmid expressing non-silencing shRNA sequence. ECA109 cells were transfected with pSi-STAT3 or pSi-Scramble, respectively, for 72 h, the gene silencing effect of pSi-STAT3 was assayed using Western blotting. As shown in Fig. 1, the protein levels of STAT3 were obviously reduced to ~ 30% in pSi-STAT3 transfected cells compared with the control cells.

### Knockdown of STAT3 induced morphological changes of ECA109 cells

**Fig. 2:**
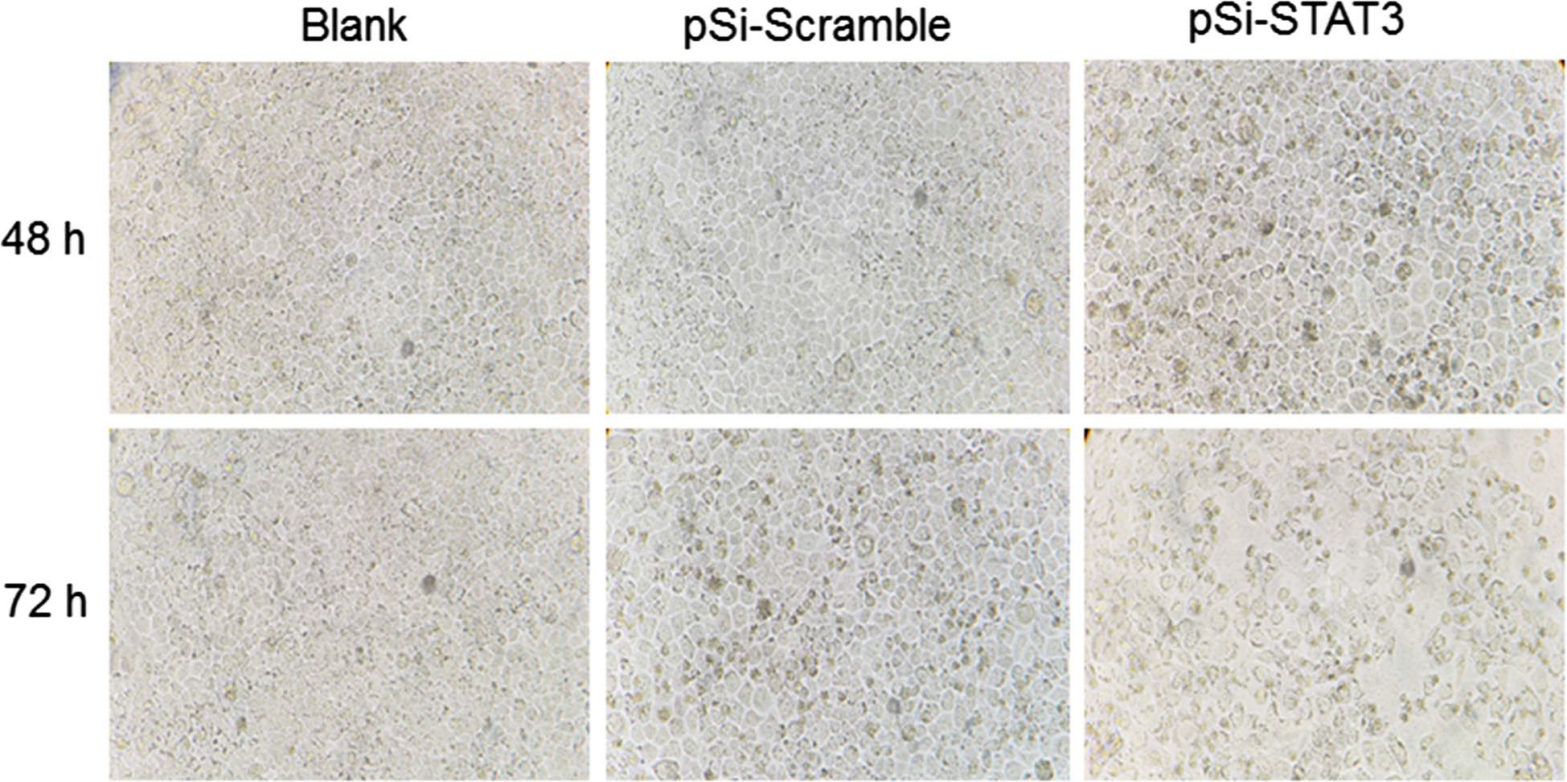
Morphological changes of ECA109 cells caused by STAT3 down-regulation. The pSi-STAT3 plasmid transfection induced apparent morphological changes like cell shrinkage, apoptotic vacuoles, membrane blebbing and forming the floating cells after transfection for 48 h and got worse after 72 h (original magnification, ×200)

Morphological changes of cells are the basic phenomena of cell apoptosis. To investigate the effects of STAT3 knockdown to the ECA109 cells, the morphological changes of cells transfected with plasmids were observed with phase-contrast microscopy. As shown in Fig. 2, we could see that the pSi-STAT3 plasmid induced apparent morphological changes like cell shrinkage, apoptotic vacuoles, membrane blebbing and forming the floating cells which appeared after transfection for 48 h and got worse after 72 h. These indicated that knockdown of STAT3 could induce morphological changes of the ECA109 cells in a time-dependent manner. Conversely, the control cells transfected with pSi-Scramble and the untreated cells displayed normal morphology with polygonal shape, distinct cell borders and intercellular tight junction all the while.

### Lack of STAT3 led to mitochondrial depolarization in ECA109 cells

**Fig. 3:**
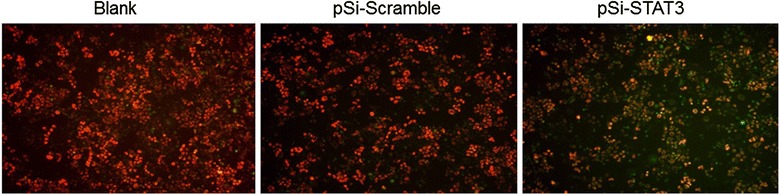
STAT3 knockdown induced loss of mitochondrial membrane potential in ECA109 cells. The cells were stained with JC-1 dye and visualized under a fluorescence microscope 24 h after transfection. The ECA109 cells in the pSi-Scramble group and untreated group exhibited red cell staining which suggested that they had normal high membrane potentials. While, pSi-STAT3 treatment caused a significant loss of JC-1 red fluorescence, that is, loss of mitochondrial membrane potential after transfected with pSi-STAT3 for 24 h, which correlated to apoptosis (original magnification, ×200)

JC-1 is a cationic mitochondrial vital dye exhibiting potential-dependent accumulation in mitochondria, as represented by a shift of fluorescence emission from red in normal polarized mitochondria to green in abnormal depolarized mitochondria. The ECA109 cells in the pSi-Scramble group and untreated group exhibited red cell staining which suggested that they had normal high membrane potentials. While, pSi-STAT3 treatment caused a significant loss of JC-1 red fluorescence, that is, loss of mitochondrial membrane potential after transfected with pSi-STAT3 for 24 h, which correlated to apoptosis (Fig. 3). The mitochondrial depolarization of ECA109 cells implied that STAT3 down-regulation caused early stage apoptosis.

### Inhibition of STAT3 expression triggered cell apoptosis in ECA109 cells

**Fig. 4:**
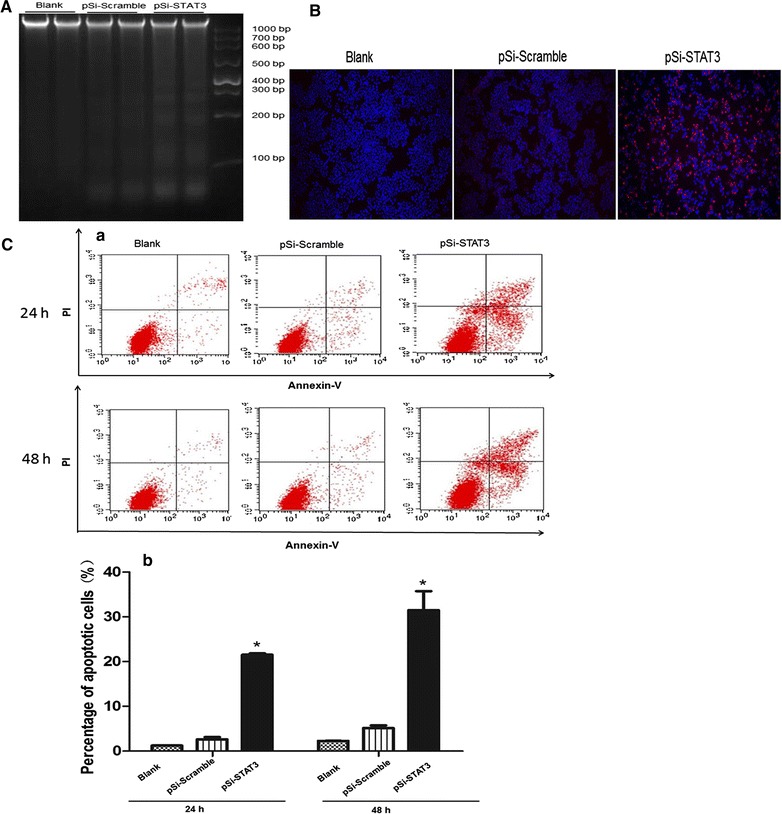
Knockdown STAT3 induced apoptosis in ECA109 cells. **A** Induction of DNA fragmentation in ECA109 cells transfected with pSi-STAT3 and pSi-Scramble. Extracted the DNA of ECA109 cells in the pSi-STAT3, pSi-Scramble and untreated groups, then analyzed with gel electrophoresis. DNA markers and sizes in base pair (bp) are indicated. **B** Detection of apoptosis in pSi-STAT3 transfected ECA109 cells was performed by TUNEL assay. The TUNEL-positive cells are indicted by a red fluorescence signal and the nuclei of normal cells are stained with DAPI in blue (original magnification, ×200). **C** Quantification of STAT3 knockdown induced apoptosis of ECA109 cells by flow cytometry. (a) ECA109 cells were transfected with pSi-Scramble or pSi-STAT3, respectively, and apoptosis rates were evaluated by flow cytometry using annexin-V and PI staining 24 and 48 h after transfection. (b) The percentage of apoptotic cells was presented as the mean ± SE.*P < 0.05, pSi-STAT3 group vs. pSi-Scramble group

The agarose gel electrophoresis of genomic DNA ladder and TUNEL assay were also performed to determine whether STAT3 down-regulation is involved in apoptosis of ECA109 cells. As shown in Fig. 4A, there was a typical pattern of DNA fragmentation like ladder appeared in ECA109 cells transfected with pSi-STAT3 but not in the pSi-Scramble and untreated groups. Consistently, pSi-STAT3 transfection significantly increased the number of TUNEL-positive apoptotic cells, but few or no apoptotic cells were seen in the pSi-Scramble treated or untreated groups (Fig. 4B). To further investigate the apoptosis of ECA109 cells induced by pSi-STAT3, we quantified the apoptosis cells by a flow cytometer using annexin-V and propidium iodide (PI) double-staining. Annexin-V can be detected in both the early and late stages of apoptosis. PI enters the cells in late apoptosis or necrosis. As shown in Fig. 4C, the percentage of apoptotic cells in the pSi-STAT3 group exhibited a significant rise compare with the pSi-Scramble group from (2.63 ± 0.47)% to (21.5 ± 0.33)% after 24 h of transfection. Further, the percentage was increased to (35.75 ± 4.28)% in the pSi-STAT3 group compared with (5.45 ± 0.61)% in the pSi-Scramble after 48 h of transfection. The untreated group, meanwhile, exhibited a low percentage of apoptotic at both times which was (1.21 ± 0.02)% at 24 h and (2.25 ± 0.09)% at 48 h. Based on the results, we assured that STAT3 down-regulation induced the apoptosis of ECA109 cells.

### Inhibition of STAT3 induced cell cycle arrest in ECA109 cells

**Fig. 5:**
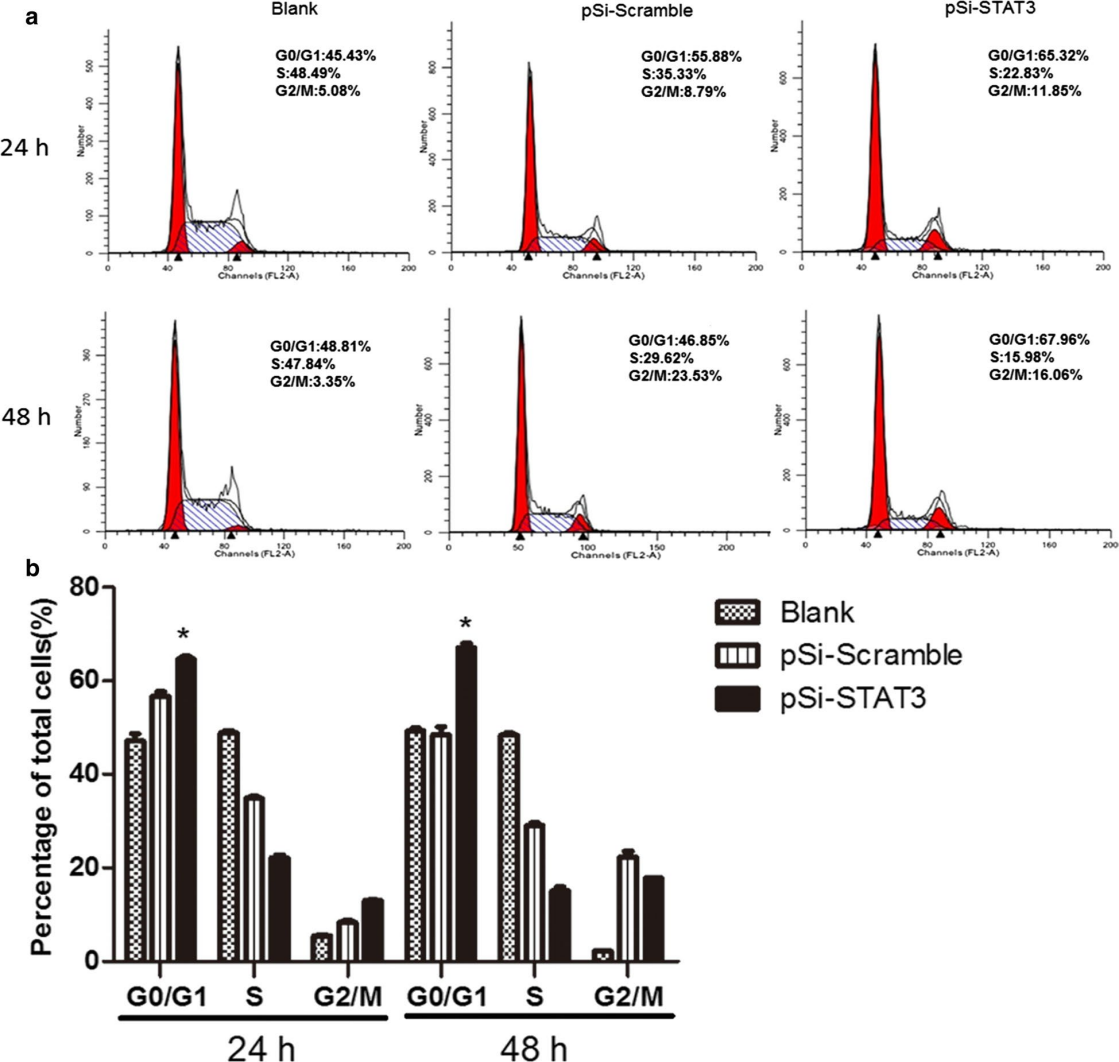
Effects of STAT3 inhibition on cell cycle distribution of ECA109 cells. **a** The cell cycles distribution of ECA109 cells in the pSi-STAT3, pSi-Scramble and untreated groups were measured with flow cytometry 24 and 48 h after transfection. **b** Representative quantitative chart depicts cell cycle phase presented as the mean ± SE.*P < 0.05, pSi-STAT3 group vs. pSi-Scramble

Cell cycle progression is a highly-ordered and tightly-regulated process which involves multiple checkpoints that assess extracellular growth signals, cell size and DNA integrity, hence the dysregulation of the cell cycle is one of the most frequent alteration during tumor development. To explore whether STAT3 down-regulation induced inhibition of cell growth had relation to the abnormal control of cell cycle, we evaluated the distribution of cell cycle by flow cytometry. As shown in Fig. 5, the percentage of ECA109 cells at G0/G1 phase was significantly higher in the pSi-STAT3 group than that in the pSi-Scramble group both 24 h and 48 h after transfection, (64.76 ± 0.57)% vs. (56.75 ± 0.88)% and (67.02 ± 0.94)% vs. (48.51 ± 0.65)%, respectively (Fig. 5). While, the untreated group and pSi-Scramble group showed no significant difference. Consequently, we confirmed that the inhibition of STAT3 had impact on the cell cycles of ECA109 cells, with the cell cycles being arrested at the G1 phase.

### Down-regulation of STAT3 in ECA109 cells inhibited cell migration

**Fig. 6:**
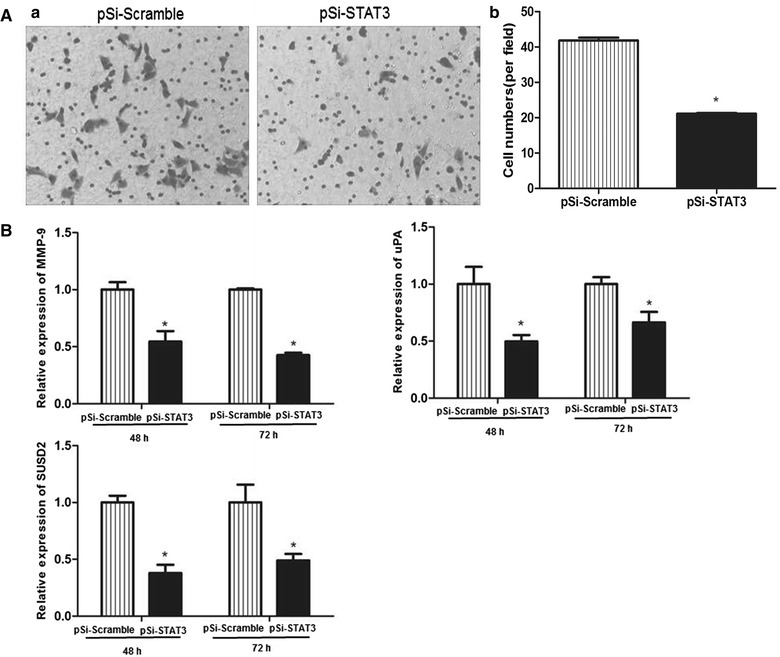
Effect of STAT3 down-regulation on cell migration in ECA109 cells. **A** Cell migration was determined by transwell assay after transfected for 72 h. The numbers of migration cells in five random microscopic fields (original magnification, ×200) were counted for each group. The results are an average of three independent experiments. **B** The mRNA relative abundance of MMP-9, uPA and SUSD2 were analysed by real-time PCR. Data shown represent the mean ± SE from three independent experiments.*P < 0.05, pSi-STAT3 group vs. pSi-Scramble

To further identify if the STAT3 down-regulation had effect on the migration of ECA109 cells, we detected the cell migration ability using transwell system assay. It was found that the migration was also decreased in the pSi-STAT3 group compared to the pSi-Scramble group after the cells transfected for 72 h (Fig. 6A). The real-time PCR analysis revealed that the mRNA levels of matrix metallopeptidase 9 (MMP-9), plasminogen activator urokinase (uPA) and sushi domain containing 2 (SUSD2) that related to migration and cancer progression were significantly reduced in the pSi-STAT3 group, compared with the pSi-Scramble group after 72 h of transfection (Fig. 6B). The consequences of transwell system assay and the real-time PCR analysis of genes related with migration and invasion demonstrated that the decline of ECA109 cells migration ability may correlate to the lack of STAT3.

### Knockdown of STAT3 in ECA109 cells affected levels of apoptosis and cell cycle-related proteins

**Fig. 7:**
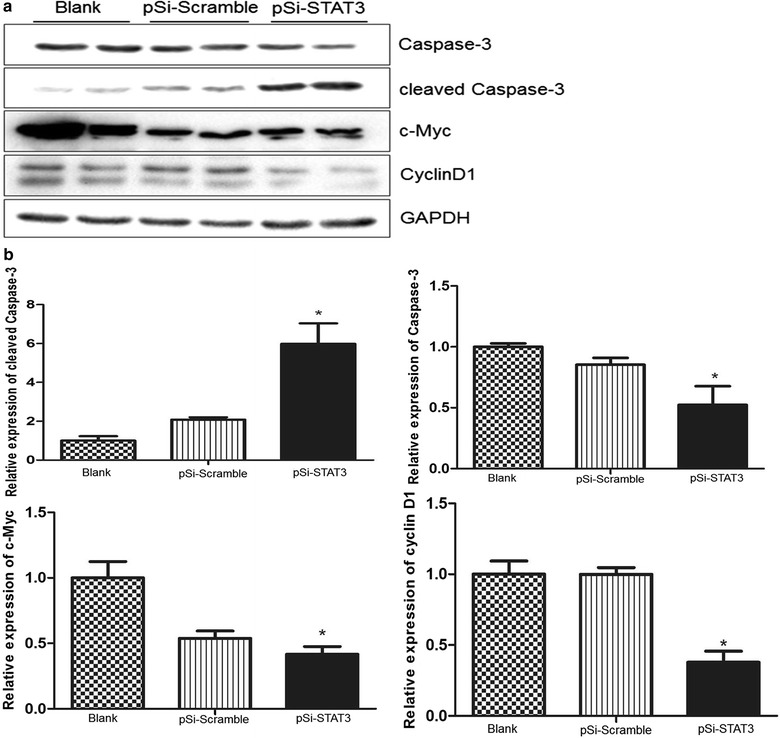
Down-regulation of STAT3 regulates the expression of apoptotic and cell cycle related proteins in ECA109 cells. **a** Cyclin D1, c-Myc, Caspase-3, cleaved Caspase-3 proteins were analyzed by Western blotting in ECA109 cells after transfection with pSi-Scramble and pSi-STAT3. GAPDH was used as loading control. **b** Quantitative levels of aforementioned proteins against GAPDH. All data are represented as the mean ± SE, *P < 0.5, pSi-STAT3 group vs. pSi-Scramble group

It has been found that cancer cells harboring aberrant STAT3 activity had elevated levels of anti-apoptotic and cell cycle regulating proteins like cyclin D1 and c-Myc. In this study, the protein levels of Caspase-3, cleaved Caspase-3, c-Myc which related to apoptosis were further assessed. As shown in Fig. 7, the levels of c-Myc and Caspase-3 were significantly decreased in ECA109 cell transfected with pSi-STAT3 contrast to that in the pSi-Scramble group. Conversely, the level of cleaved Caspase-3 was increased markedly in the pSi-STAT3 group than that in the pSi-Scramble group. Cyclin D1, a G0/G1 phase associated protein, was also decreased in the pSi-STAT3 group compared to the pSi-Scramble group, which was consistent with the cell cycle analysis.

## Discussion

Esophageal cancer is a serious malignancy and a major cause of cancer-related deaths worldwide. Current treatment options include surgery, radiation and chemotherapy. But the prognosis of esophageal cancer is usually poor, even though the tumor was surgically removed at early stage. Thus, early diagnosis and targeted therapy are critical. Herein, we investigated the functional effects of STAT3 down-regulation in ECA109 to illustrate the role of STAT3 in esophageal cancer.

STAT3 is overexpressed in a variety of human malignancies. Persistent STAT3 activation had been described in various types of solid and hematological cancers. For instance, activation of STAT3 by immune cells and cancer-associated fibroblasts had been shown to be essential in colorectal cancer [18]. Moreover, in human prostate tumor tissues, androgen receptor down-regulation further leads to the development of prostate cancer stem-like cells, which requires STAT3 activity [19]. In addition, the HER2-STAT3 network has been shown to contribute to the aggressive phenotype of breast cancer stem cells [20], and the JAK2/STAT3 signaling pathway is required for the growth of CD44^+^CD24^−^ stem cell-like breast cancer cells in human tumors [21]. Further, studies also placed STAT3 at a central node in esophageal cancer. For example, over-expression of miR-124 induced inhibition of cell proliferation, block of G1/S phase transition and cell apoptosis in esophageal cancer by down-regulated the mRNA and protein levels of STAT3 [22] and the tumor suppressor liver kinase B1 could inhibit esophageal cancer cell proliferation through suppression of STAT3 transaction [23].

Constitutive activation of STAT3 is involved in many cellular processes. Blocking STAT3 in human tumor cells has been shown to down-regulate Mcl-2 expression and induce tumor cell apoptosis [24], and metformin promoted autophagy and apoptosis in esophageal squamous cell carcinoma by down-regulating STAT3 signaling [25] indicated that active STAT3 contributed to the inhibition of tumor apoptosis. In present study, STAT3-specific shRNA was used to inhibit STAT3 signaling, and found that there were significant morphological changes on the esophageal cancer ECA109 cells transfected with pSi-STAT3, like shrinkage and deformation, increase of round and detached cells, which are the basic characteristics of apoptotic cells. And further analysis with mitochondrial membrane potential, DNA ladder analysis, TUNEL assay suggested that reduction of STAT3 gene expression could cause significant apoptosis of ECA109 cells. Then we quantified the apoptosis cells by a flow cytometer and found the percentage of apoptotic cells in the pSi-STAT3 group was significantly increased compared with that in the pSi-Scramble group, and consistent with the significant increase cleaved Caspase-3 after STAT3 down-regulation.

The proliferation of eukaryotic cells is controlled at specific stages of the cell cycle, particularly at the Gl to S and G2 to M transition and affected through the periodic activation and inactivation of a series of CDKs. The checkpoints of G1/S are located at the G1 phase of the cell cycle. The D-type cyclins (cyclin D1, D2, D3) are synthesized earlier in G1-phase and form complex with CDKs to regulate the G1 to S cell transition [26]. As previously described, when STAT3 was phosphorylated, it could form dimers and move from the cytoplasm to the nucleus stimulating transcription of STAT3 target genes, including cyclin D1, c-Myc which correlated with cell cycle, and vascular endothelial growth factor [27]. From our consequence, inhibition of STAT3 induced G0/G1 cell cycle arrest with inhibition of cyclin D1 gene expression, which showed that STAT3 may regulate cell cycle of ECA109 cell through cyclin D1. But the interesting thing is that our former study showed a G2/M arrest with cyclin B1 down-regulation when the STAT3 was knockdown in TE1 cell line which is also an esophageal squamous cell carcinoma [28]. Few studies had demonstrated this uncommon difference that the same cytokine could induce different cell cycle arrests in the same cancer [9, 29], which may because of the different mode of inhibition or tyrosine kinases activity.

The invasion and metastasis characteristic of esophageal cancer is not only the postoperative recurrent nature, but also causes many patients to lose the operation opportunity, and the migration is the first step of tumor metastasis. Thus, it is important to identify the factors that correlate to the cancer migration. Matrix metalloproteinases (MMPs) are overexpressed in almost all human cancers [30, 31]. The matrix metalloproteinase-2 (MMP-2), MMP-9 and urokinase-PA (u-PA) are responsible for the degradation of extracellular matrix components and play important roles in the process of cancer invasion and metastasis [32–34]. And as a novel molecule related with cancer migration, there were various phenotype assays indicated that Sushi Domain Containing 2 (SUSD2) increases the migration of breast cancer cells [35]. In our study, we examined the migration ability of ECA109 cells using transwell chambers, and detected the migration related genes like MMP-9, uPA and SUSD2, and the genes were significantly decreased by pSi-STAT3 transfection, which indicated that the migration ability of ECA109 cells was declined by STAT3 down-regulation. Further, all the aforementioned genes are also related to tumor invasion and metastasis, we presumed that the STAT3 knockdown may inhibit the invasion and metastasis of ECA109 cells as well. Lastly, we also note that this study has some limitations requiring attention. The possible effects that caused by STAT3 down-regulation in vivo should be detected in further studies.

## Conclusions

Based on our results, we concluded that down-regulation of STAT3 could induce the apoptosis and G1 cell cycle arrest in esophageal carcinoma ECA109 cells, and also inhibit the migration ability of cells. In summary, our study presents several evidences to support STAT3 as a potential target for esophageal cancer therapy.

## Abbreviations

STAT3: signal transducer and activator of transcription 3
shRNA: small hairpin RNA
qRT-PCR: quantitative real-time polymerase chain reaction
MMP-9: matrix metalloproteinases-9
SUSD2: sushi domain containing 2
uPA: urokinase type plasminogen activator
JAK: Janus kinase
INFα: interferon-α
IL-6: intereukin-6
EGF: epidermal growth factor
HGF: hepatocyte growth factor
GAPDH: glyceraldehyde 3-phosphate dehydrogenase
SDS-PAGE: sodium dodecyl sulfate polyacrylamide
